# Temperature Increase Exacerbates Apoptotic Neuronal Death in Chemically-Induced Ischemia

**DOI:** 10.1371/journal.pone.0068796

**Published:** 2013-07-08

**Authors:** Chunyan He, Ann Stroink, Laura Vogel, Chen Xu Wang

**Affiliations:** 1 School of Biological Science, Illinois State University, Normal, Illinois, United States of America; 2 Central Illinois Neuroscience Foundation, Bloomington, Illinois, United States of America; Massachusetts Eye & Ear Infirmary, Harvard Medical School, United States of America

## Abstract

It is well-established that hyperthermia increases neuronal death and worsens stroke outcome. However, little is known about the mechanisms of how hyperthermia is involved in this neuronal death process. In the present study, we examined how temperature increase exacerbates neuronal death using a model of chemical ischemia. Chemical ischemia was induced by treating SH-SY5Y neuroblastoma cells with sodium azide and deoxyglucose. Temperature increase was treated by placing the cells at 37°C (control) and 41°C (experimental). Cell survival was determined by trypan blue assay and ATP levels were measured with ATP assay kits. Protein expression was detected by western blot. Treatment with sodium azide resulted in cell death in a dose-responsive manner. Increased temperature worsened the ATP depletion and cell volume shrinkage. Temperature increase also enhanced ER stress as demonstrated by the elevated level of phospho-eIF2α and C/EBP homologous protein (CHOP). Inhibition of CHOP expression significantly decreased sodium azide-induced neuronal death. In addition, the increased temperature intensified the activation of caspase-3, an apoptotic effector protease, and inhibition of capspase-3 significantly reduced cell death. These findings support that temperature increase worsened the neuronal death by depleting intracellular ATP, inducing ER stress response and activating apoptotic signal transduction.

## Introduction

Stroke causes neuron death and brain tissue damage, and many complications after stroke adversely affect outcomes. Hyperthermia, a common complication occurring in 50% patients within 48 hours following an ischemic insult [1], negatively correlates with the final outcome of stroke. Hyperthermia has adverse effects on treatment regimens that work under normothermic conditions [2], [3]. For example, hyperthermia abolishes the therapeutic actions of thrombolytic treatment with tissue plasminogen activator, the only effective pharmacological treatment for stroke patients. We have studied hyperthermia and ischemic brain injury using an embolic model in rats. Findings from these studies demonstrate that hyperthermia significantly exacerbates neuronal death, increases infarct volume and worsens mortality [4], [5]. These findings support that hyperthermia is neuro-destructive in ischemic brain injury.

During cerebral ischemia, most cell death in the ischemic core is necrotic while apoptotic death is observed in the penumbra, the region surrounding the ischemic core [6]. There are two major signaling pathways that control the initiation of apoptosis: the extrinsic and intrinsic pathways. The extrinsic pathway involves death receptors and caspase-8-initiated activation of caspase-3, the apoptotic effector protease. The intrinsic pathway is mitochondrial dependent, induced by a wide range of death stimuli such as DNA damage, hypoxia and withdrawal of growth factors, resulting in the mitochondrial release of cytochrome c, which mediates activation of caspase-9 and caspase-3 [7], [8]. Activated caspase-3 cleaves and inactivates PARP-1, which abolishes PARP-1 activity in repair of DNA damage [9].

Besides the two classic signaling pathways, studies have demonstrated that endoplasmic reticulum (ER) stress activation also causes apoptosis in ischemic injury [10], [11]. Eukaryotic cells evolve a set of molecular pathways for coping with ER stress, collectively termed the unfolded protein response (UPR). The UPR is activated when cells are under stress and is carried out by three trans-membrane initiator proteins: PERK (protein kinase RNA (PKR)-like ER kinase), IRE1 (inositol-requiring protein-1) and ATF6 (activating transcription factor-6). All three effector proteins bind to the ER chaperone, Bip (GRP78), on their ER luminal domains, where Bip acts to repress their activities. However, when cells are under stress Bip dissociates from these initiator proteins, allowing the activation of these initiator proteins. All three initiator proteins mediate signaling pathways to induce the expression of C/EBP homologous protein (CHOP) [12]–[14]. Whereas UPR is originally activated to protect the cells, it becomes destructive when ER stress is sustained. PERK-eIF2α is the first identified molecular mechanism in the UPR process and activation of this signal transduction pathway is involved in ischemic neuronal death [15], [16].

Although it is well-established that temperature increase worsens stroke outcome, the mechanisms by which this process occurs are still not very clear [3], [17]. The present study was designed to test the hypothesis that temperature increase regulates the signal transduction pathways during ischemic cell death. Specifically, we examined whether temperature increase exacerbates neuronal death by regulating intracellular ATP depletion, ER stress and caspase activation, using a model of chemical ischemia.

## Methods

### Materials

All chemicals were purchased from Sigma-Aldrich (St. Louis, USA), except where indicated.

### Cell Culture

Human SH-SY5Y neuroblastoma cell line [18] (kindly provided by Dr. J. X. Comella, Lleida, Spain) was grown in Dulbecco's Modified Eagle's Medium (DMEM) supplemented with penicillin (100 units/ml), streptomycin (100 ug/ml) (Fisher Scientific, Pittsburg, USA), and 10% (vol/vol) heat-inactivated fetal calf serum (Atlanta Biologicals, Lawrenceville, USA) at 37°C in a saturated humidity atmosphere containing 95% air and 5% CO_2_.

### Chemical Ischemia

In order to study the signaling events of neuronal death in response to ischemic injury, we adapted a model of chemical ischemia in SH-SY5Y cells, as described previously [19]. In brief, culture medium was removed and the cells were washed with PBS two times. Thereafter, ischemic solution containing 0.5 mM 2-d-deoxyglucose (2-DG) and different concentrations of sodium azide was added into the cell culture. In this model, oxidative ATP production was blocked with sodium azide, and glycolytic ATP production was impeded by removing glucose and preventing metabolism of retained intracellular glucose with 2-DG. Chemical ischemia induced by sodium azide has been used in other types of cells, including C2C12, HEK293 and PC12 [20].

### Induction of Temperature Increase

Treated and control cells were transferred into pre-heated CO_2_ incubators set at either 37°C or 41°C. The 41°C was chosen for the treatment of increased temperature since our preliminary studies showed that this temperature produced consistent results in cell death induction. The cells were kept at these temperatures for a predetermined period of time and then collected for analysis.

### Trypan Blue Assay

Cell viability was assessed by trypan blue exclusion assay [18]. In brief, 500 µl of 0.4% trypan blue solution was added to 500 µl cell suspension in culture medium. The suspension was gently mixed and applied to hemacytometer. Viable and dead cells were identified and counted under light microscope. Blue cells failing to exclude dyes were considered nonviable, and transparent cells were considered viable. The percentage of viable cells was calculated on the basis of total number of cells (viable plus nonviable).

### Flow Cytometry

Cells were collected and re-suspended in DMEM with a density of 1×10^6^ per ml after the treatments. Cell size was measured by BD FACScan flow cytometer with an aid of Cell Quest Pro software (BD Biosciences, San Jose, USA). In flow cytometry, forward scatter (FSC) is a parameter that measures the light scattered and is proportional to the cellular volume. The decreased forward scatter indicates decreased cellular volume. The data was acquired using FCS with voltage E00. A total of 50,000 cells were analyzed per sample.

### Annexin V-FITC/Propidium Iodide (PI) Staining

Equal amount of cells were grown on microscope cover glasses (diameter 1.5 cm). After treatments, cells were stained with Annexin V-FITC and PI kit (Invitrogen, Grand Island, USA. cat #: 13242) according to the manufacturer’s protocol. Briefly, staining solution including Annexin V-FITC and PI was added to the cover glass and incubated for 15 minutes. The cover glass was then mounted onto microscope slide and examined under a light microscope equipped with appropriate filters. Apoptotic cells stained with Annexin V-FITC show green fluorescence and necrotic cells stained with both Annexin V-FITC and PI show red fluorescence as well as green fluorescence.

### ATP Assay

Cells were grown in 96-well plates. After the treatments, intracellular ATP was measured with Enzylight™ ATP Assay Kit (Bioassay System, Hayward, USA. cat #: EATP-100) according to manufacturer’s protocol. ATP in the culture medium was measured with ENLITEN ATP Assay System, as described previously [21] (Promega, Madison, USA. cat #: FF2000).

### Western Blot

Western blot analyses were carried out as described previously [18], [22]. In brief, cells were harvested and lysed in cold lysis buffer [50 mM Tris (pH 7.4), 150 mM NaCl, 2 mM EDTA, 10% glycerol, 1% Triton X-100, 1 mM PMSF and 2% protease inhibitor mixture]. The cell lysates were centrifuged for 15 minutes at 18,000×*g* at 4°C, supernatants were collected and protein concentrations in the supernatants were determined using Bio-Rad protein assay (Bio-Rad Laboratories, Hercules, USA). The cell lysates with equal amount of proteins were subjected to electrophoresis on 12% SDS-polyacrylamide gels and transferred to polyvinylidene difluoride membrane. The membranes were blocked in TBS-T (Tris-buffered saline with 0.05% Tween-20) containing 5% milk and blotted overnight with various primary antibodies. The membranes were washed and incubated for 1 hour with horseradish peroxidase (HRP) - conjugated secondary antibodies (Jackson ImmunoResearch, West Grove, USA). The membranes were washed and developed by chemiluminescence (Fisher Scientific, Pittsburg, USA). The primary antibodies include anti-caspase-3 (Cell Signaling Technology, Danvers, USA. cat #: 9662, dilution 1∶1000), anti-CHOP (Cell Signaling. cat #: 2895, dilution 1∶1000), anti-eIF2α (Cell Signaling. cat #: 5324, dilution 1∶1000), anti-phospho-eIF2α (Cell Signaling. cat #: 3597, dilution 1∶1000), anti-PERK (Cell Signaling. cat #: 3192, dilution 1∶1000), anti-PARP-1 (Cell Signaling. cat #: 9532, dilution 1∶1000), anti-Bip (Cell Signaling. cat #: 3177, dilution 1∶1000) and anti-β-actin (Santa Cruz Biotechnology, Santa Cruz, USA. cat #: 130657, dilution 1∶5000).

### Caspase-3 Activities

Caspase-3 activities were measured with EnzChek assay kit according to manufacturer’s protocol, using rhodamine 110 bis [N-CBZ-L-aspartyl-L-glutamyl-L-valyl-L-aspartic amide (z-DEVD-R110)] as a substrate (Life Technologies, Grand Island, USA. cat #: E13184). Upon enzymatic cleavage, this non-fluorescent bisamide substrate was converted to fluorescent monoamide and R110. Caspase-3 inhibitor z-DEVD-FMK (BD Pharmingen, San Diego, USA) was dissolved in DMSO. Cells were treated with either DMSO or z-DEVD-FMK diluted in DMSO (20 µM) for 2 hours before the experiment [23], [24]. After the treatments, cells were then collected, lysed and caspase-3 activities were assayed [25].

### shRNA Transfection

Cells grown in 6-well plate were transfected when cells reached around 50% confluence. Transfection was performed by adding lentiviral particles encoding CHOP shRNA as well as polybrene (Santa Cruz Biotechnology. cat #: 35437-V) to culture medium. After 16 hours transfection, the medium was replaced with DMEM containing 5% FBS. Two days later, puromycin dihydrochloride at a concentration of 2 µg/ml was added to the medium for selection. Cells that were not successfully transfected with the lentiviral particles were killed by puromycin dihydrochloride. The inhibition of CHOP expression was confirmed by western blot analysis. The control cells received mock transfection with lentiviral particles encoding copGFP (Santa Cruz Biotechnology. cat #: 108084).

### Statistical Analyses

Data of cell survival, ATP levels and caspase-3 activities were expressed as mean ± SEM, and were analyzed with one-way analysis of variance (ANOVA) followed by Tukey post hoc test when more than two groups were compared. For comparison between two groups, a t-test was used. The interaction between temperature and chemical ischemia was analyzed with a two-way ANOVA. A *p* value less than 0.05 was considered statistically significant.

## Results

### Temperature Increase Enhances Neuronal Cell Death

**Figure 1:**
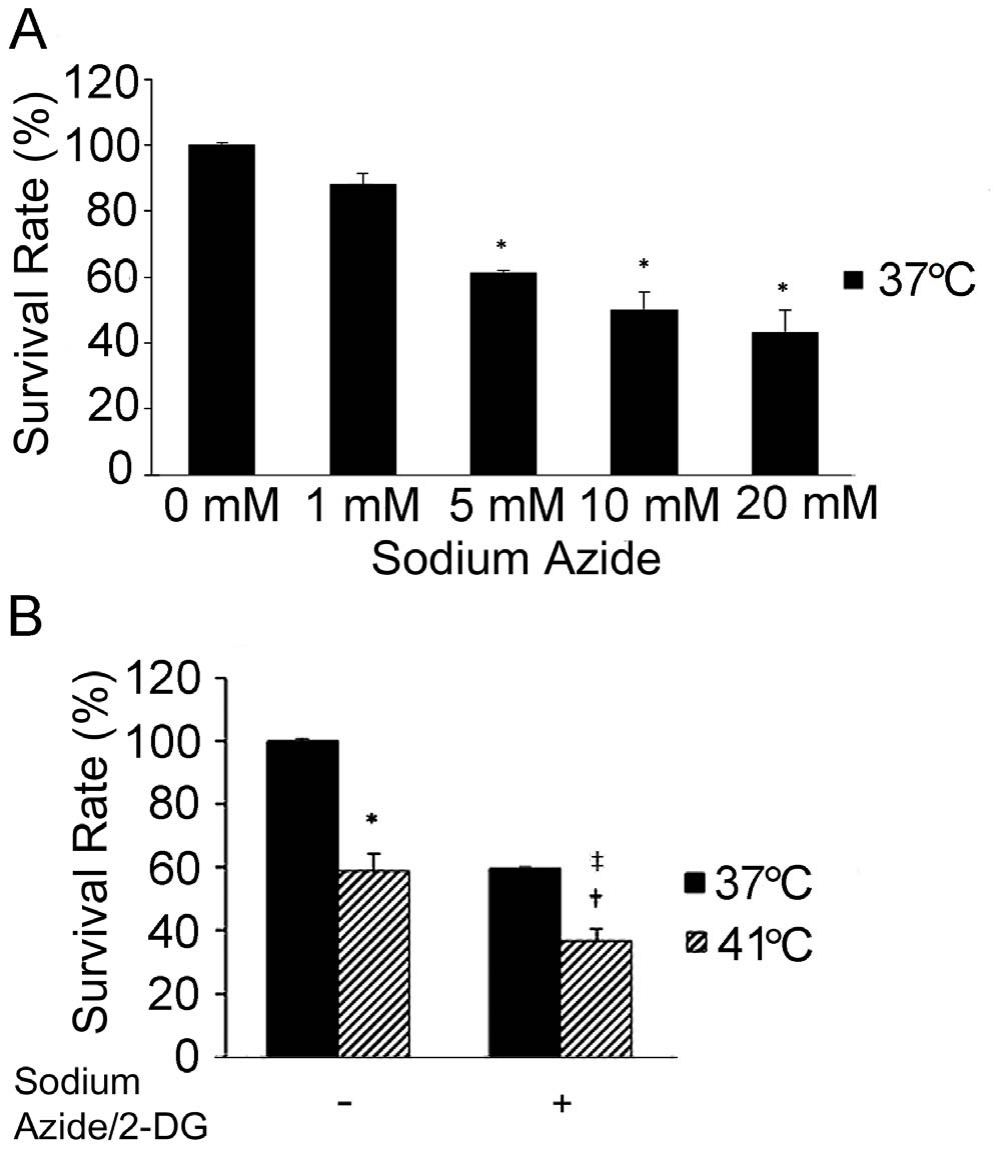
Temperature increase exacerbated sodium azide/2-DG-induced cell death. (A) Sodium azide caused dose-dependent cell death. Neuronal death was induced with different concentrations of sodium azide plus 0.5 mM 2-DG for a period of 1 hour. The time point of cell collection for the analysis was decided based on the findings from our preliminary studies. Cell survival was detected with trypan blue exclusion assay. Error bars represent mean ± SEM. n = 4. * denotes significant difference from 37°C control cell (0 mM) (*p*<0.01). (B) Increased temperature resulted in more cell death. Cells were treated as indicated for a period of 1 hour and cell survival was detected with trypan blue exclusion assay. * denotes significant difference from 37°C control (*p*<0.01). † denotes significant difference from 37°C with sodium azide/2-DG (*p*<0.01). ‡ denotes significant difference from 41°C without sodium azide/2-DG (*p*<0.05).

Neuronal death was induced by treatment of SH-SY5Y cells with different concentrations of sodium azide and 0.5 mM 2-DG (Figure 1A). The cells were collected 1 hour later for the analysis. Treatment with sodium azide caused a dose-dependent reduction of cell viability at 37°C. When compared to the cells treated without sodium azide (0 mM), cell viability was reduced significantly by the treatment of sodium azide at 5 mM (*p*<0.01), 10 mM (*p*<0.01) and 20 mM (*p*<0.01) respectively. However, sodium azide at 10 mM and 20 mM did not cause significantly lower cell viability than sodium azide at 5 mM. Therefore, 5 mM sodium azide and 0.5 mM 2-DG were chosen for the subsequent studies.

Compared to cells treated at 37°C, increased temperature (41°C) resulted in decreased viability in cells either without (*p*<0.01) or with sodium azide/2-DG (*p*<0.01) (Figure 1B). The cell viability was also significantly lower in the cells treated at 41°C plus sodium azide/2-DG than in the cells treated with increased temperature alone (*p*<0.05). Two-way ANOVA analyses showed a significant effect of treatment with increased temperature (*p*<0.01) and sodium azide/2-DG (*p*<0.01) on the reduction of cell survival. In addition, interaction between the increased temperature and sodium azide/2-DG was significant in reducing cell survival (*p*<0.05). Thus, the combination of increased temperature and sodium azide/2-DG caused neuronal death more efficiently than either treatment alone.

### Neuronal Death is Apoptotic

**Figure 2:**
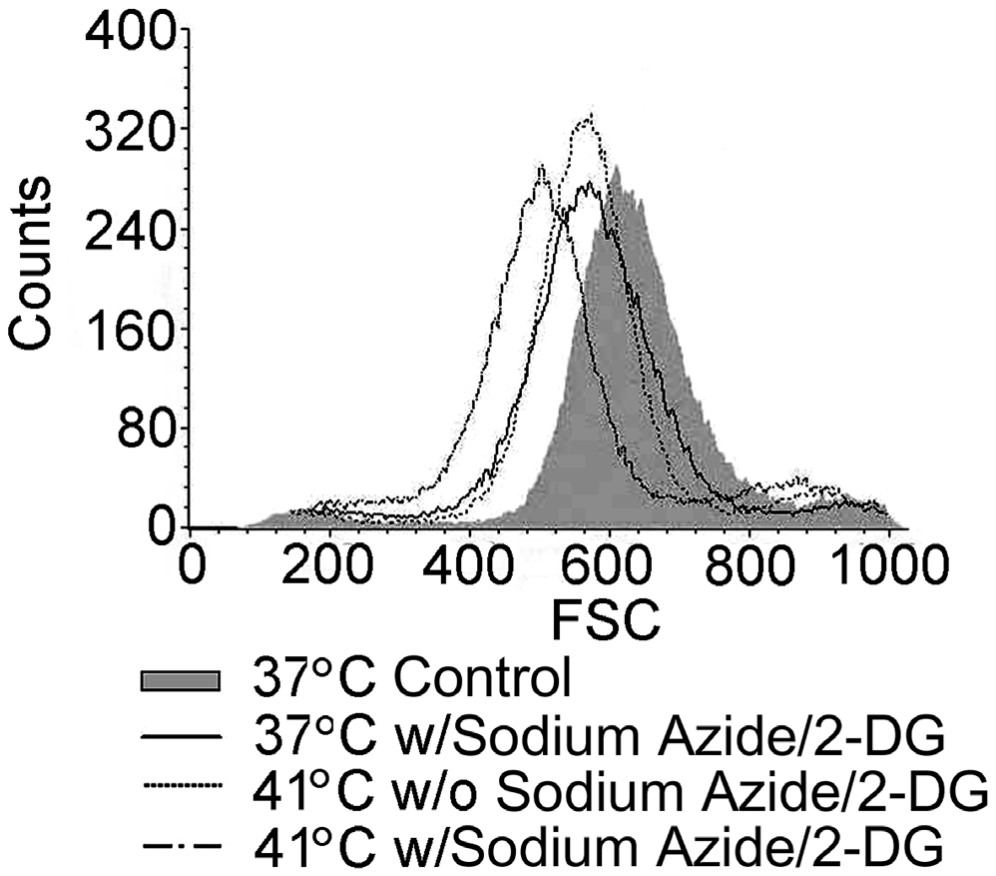
Temperature increase worsened sodium azide/2-DG induced reduction of cellular volume. Cells received different treatments for 1 hour, and then cell volume was measured by BD FACScan flow cytometer with an aid of Cell Quest Pro. The data were acquired using FCS with voltage E00. A total of 50,000 cells were analyzed per sample. Flow cytometry analysis showed smaller forward scatter value following the treatment with sodium azide/2-DG or increased temperature alone. Combination treatment of sodium azide/2-DG and increased temperature produced addictive actions in the decrease of forward scatter value. Data were representative of one of three independent experiments. w/: with; w/o: without.

As shown in Figure 2, treatment of the cells with sodium azide/2-DG at 37°C for a period of 1 hour resulted in decreased cell size (by forward light scatter) when compared to the 37°C non-treatment control. Treatment with increased temperature (41°C) for 1 hour also resulted in decreased forward scatter than the 37°C control. In addition, treatment of the cells at 41°C with sodium azide/2-DG further decreased forward scatter. These results demonstrate that treatment with sodium azide/2-DG and increased temperature resulted in shrinkage of cell volume, indicating the cell death was apoptotic.

**Figure 3:**
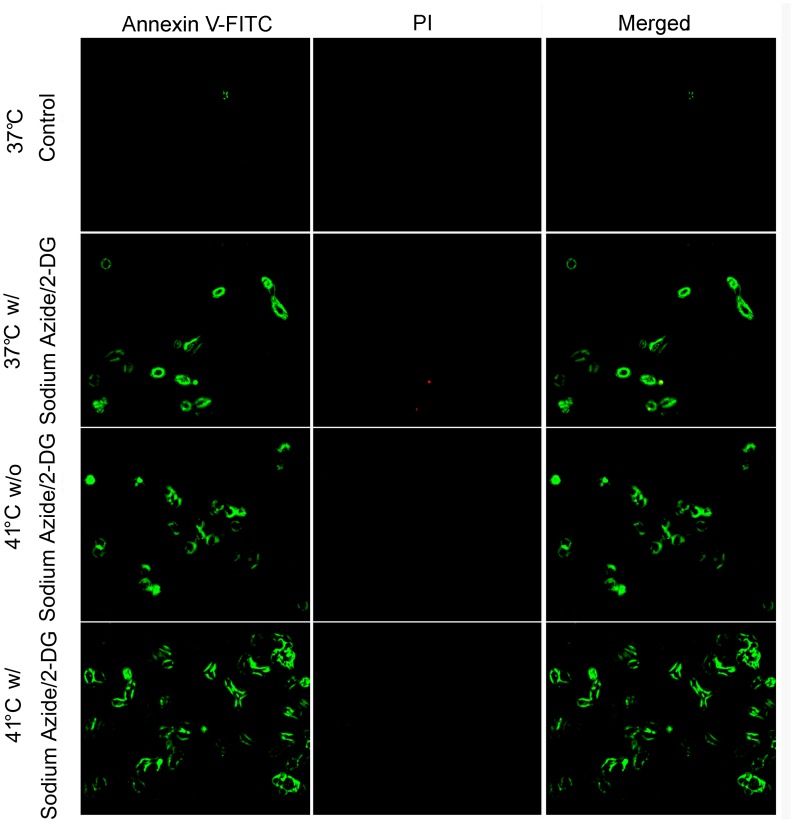
Apoptosis was detected with Annexin V-FITC and PI staining. Cells were double stained with Annexin V-FITC and PI after the treatments for 1 hour. Apoptosis with Annexin V-FITC positive staining (green fluorescence) was detected in cells treated with high temperature alone, sodium azide/2-DG alone or the combination of sodium azide/2-DG plus high temperature. Representative images were shown in the figure. w/: with; w/o: without.

To further confirm that the cell death induced by the treatments was apoptotic, cells were stained with Annexin V-FITC and PI (Figure 3). In the 37°C non-treatment control, no fluorescence signal was detected for either dye. Cells treated with either sodium azide/2-DG or temperature increase (41°C) for 1 hour exhibited green fluorescence but no red fluorescence, indicating only Annexin V-FITC staining. Cells treated with sodium azide/2-DG at 41°C for 1 hour showed more green fluorescence than in the cells treated with sodium azide/2-DG or temperature increase alone. Thus, these results further support that the cell death was apoptotic.

### Temperature Increase Aggravates ATP Depletion

**Figure 4:**
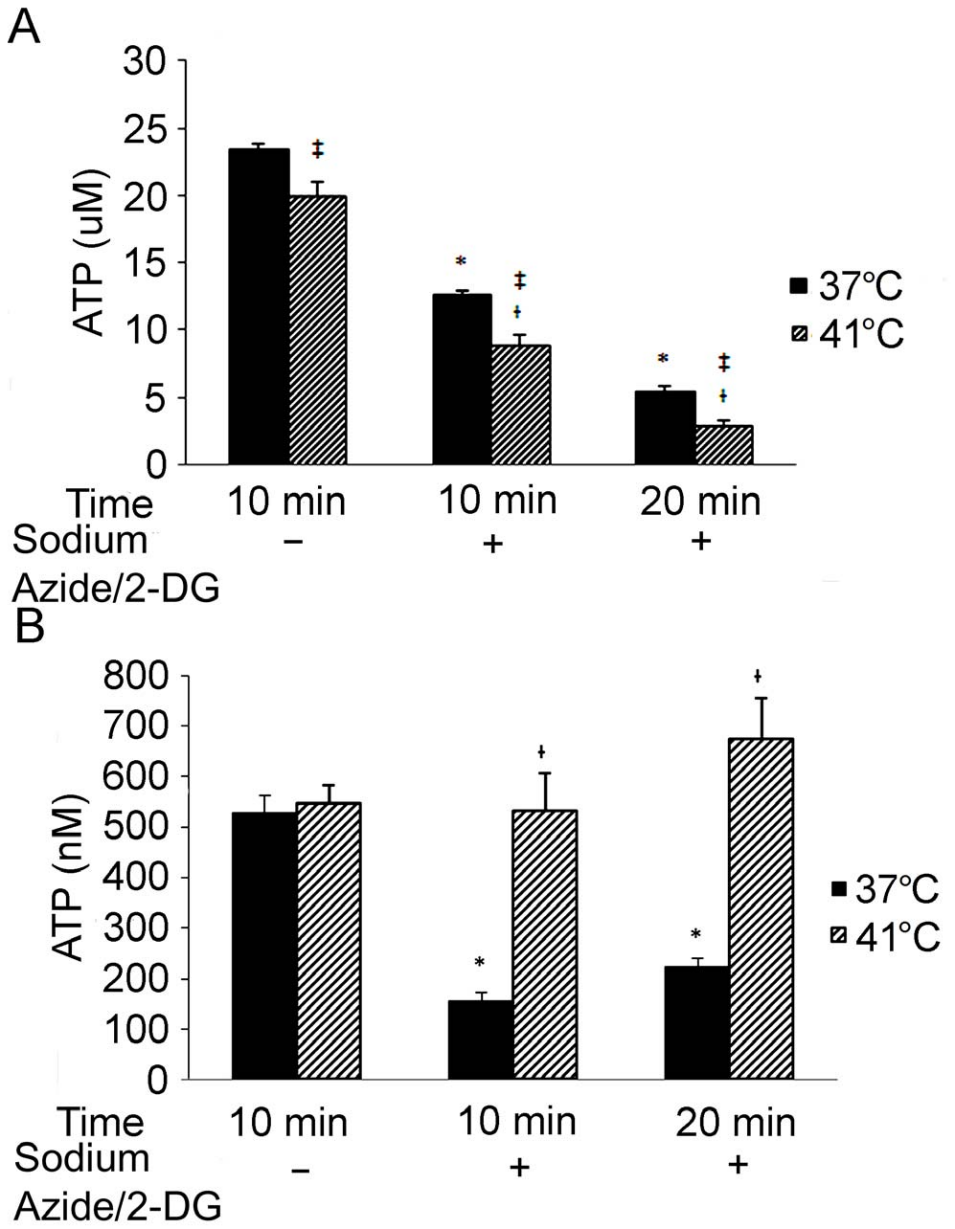
Intracellular and extracellular ATP levels changed following treatments. Neuronal death was induced by treating the cells with 0.5 mM 2-DG and 5 mM sodium azide for the indicated time. Control cells were incubated with fresh regular medium for 10 minutes. (A) Intracellular ATP was measured. Error bars represent mean ± SEM. n = 4. * denotes significant difference from 37°C controls (*p*<0.01); † denotes significant difference from 41°C control (*p*<0.01); ‡ denotes significant difference from the cells received the treatment at 37°C (*p*<0.05). (B) Extracellular ATP was measured in the culture medium following the treatments. Error bars represent mean ± SEM. n = 4. *denotes significant difference from 37°C controls (*p*<0.01); † denotes significant difference from the group received the treatment with sodium azide/2-DG at 37°C (*p*<0.01).

Compared to the non-treatment control, intracellular ATP levels dropped dramatically after the cells were incubated with sodium azide/2-DG for 10 minute (*p*<0.01) and 20 minute (*p*<0.01) at 37°C (Figure 4A). When compared to cells at 37°C, increased temperature resulted in lower ATP in the cells either without (*p*<0.05) or with sodium azide/2-DG treatment at 10 (*p*<0.05) and 20 (*p*<0.05) minutes. As compared to the 41°C control, the ATP level in the cells treated with sodium azide/2-DG for 10 (*p*<0.01) and 20 (*p*<0.01) minutes at 41°C was also significantly decreased.

Next, ATP concentrations in the cell culture medium were examined (Figure 4B). At 37°C, ATP levels at both 10 and 20 minute following the treatment with sodium azide/2-DG were significantly lower than that in the non-treatment control (*p*<0.01). However, at 41°C the ATP change following the treatment with sodium azide/2-DG was not significant as compared to the non-treatment control (*p*>0.05). In the culture medium of cells treated with sodium azide/2-DG, the ATP level at 41°C was significantly higher than that at 37°C (*p*<0.01).

### ER Apoptotic Pathway is Activated

**Figure 5:**
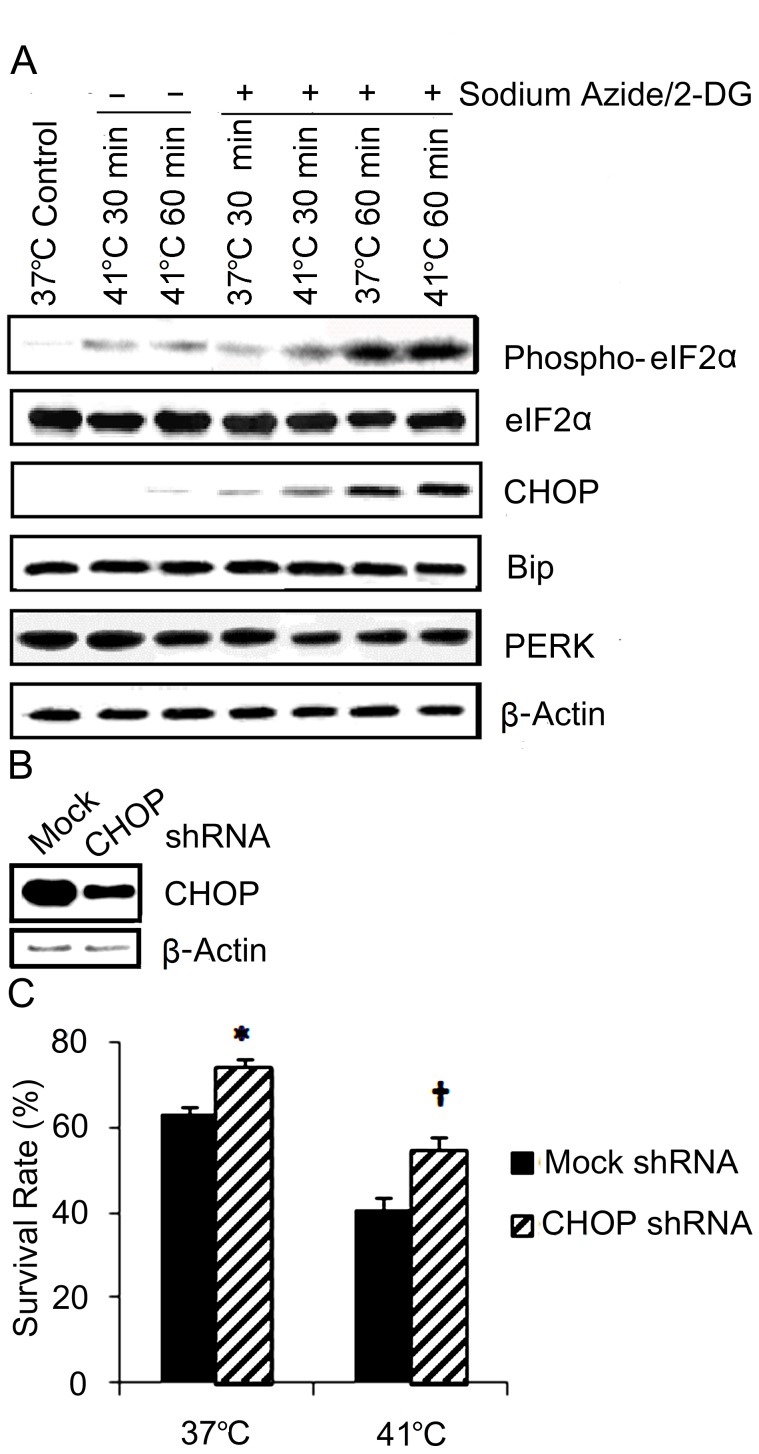
Temperature increase worsened sodium azide/2-DG induced ER stress. (A) Cells were subjected to treatments with sodium azide/2-DG, increased temperature or combination of sodium azide/2-DG plus increased temperature for a period of 30 minute or 1 hour. Control cells received no treatment. The cells were then collected and protein expressions were analyzed with western blot. β-actin was used as a loading control. (B) CHOP shRNA transfection significantly inhibited CHOP expression. SH-SY5Y cells were transfected with lentiviral particles encoding copGFP (mock) or CHOP shRNA. CHOP protein levels were analyzed by western blot. β-actin was used as a loading control. (C) CHOP shRNA transfection significantly reduced sodium azide/2-DG-induced cell death. Mock or CHOP shRNA transfected SH-SY5Y cells were treated with sodium azide/2-DG for 1 hour at 37°C and 41°C. Cell death was detected with trypan blue exclusion assay. * (p<0.05) and † (p<0.01) denote significant difference from the group received mock transfection.

The activation of the ER signal pathway was evaluated by analyzing the phospho-eIF2α and CHOP levels (Figure 5A). Western blot analyses showed phospho-eIF2α elevated in the cells following treatment with temperature increase (41°C). The combination treatment of increased temperature and sodium azide/2-DG further enhanced phosphorylation of eIF2α more than sodium azide/2-DG or increased temperature alone. The change of CHOP paralleled that of phospho-eIF2α. CHOP remarkably increased in the cells treated with sodium azide/2-DG at 37°C. Temperature increase in combination with sodium azide/2-DG further enhanced CHOP induction more than sodium azide/2-DG or temperature increase alone. However, total eIF2α, PERK and Bip did not alter significantly following these treatments. These data suggest that temperature increase worsened the ER stress induced with sodium azide/2-DG.

To study the role of ER stress pathway in the cell death, CHOP expression was inhibited by transfection of cells with shRNA, which was demonstrated by western blot analysis (Figure 5B). Then, sodium azide/2-DG-induced cell deaths were examined in these cells at 37°C and 41°C. Compared to the cells received mock transfection (GFP), transfecting the cells with CHOP shRNA significantly decreased cell death at 37°C (p<0.05), and 41°C (p<0.01) (Figure 5C).

### Caspase Inhibition Attenuates Neuronal Death

**Figure 6:**
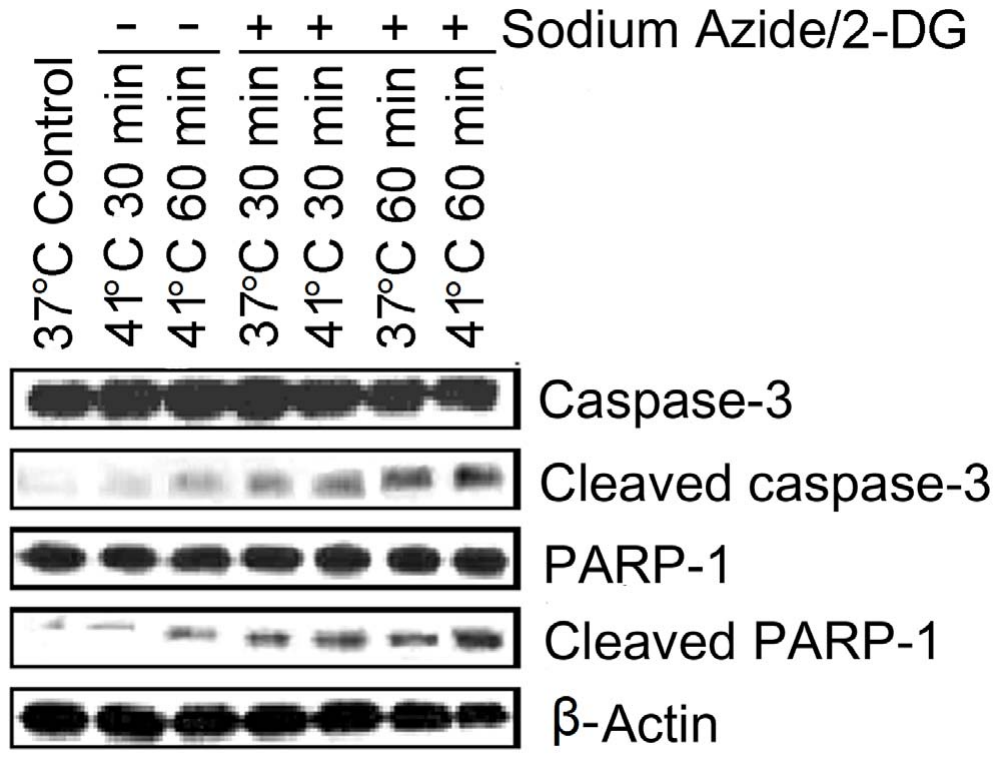
Caspase-3 was activated following the treatments. Cells were subjected to treatments with sodium azide/2-DG, increased temperature or combination of sodium azide/2-DG plus increased temperature for a period of 30 minute or 1 hour. Control cells received no treatment. Cleavage of caspase-3 and PARP-1 were analyzed with western blot. β-actin was used as a loading control.

In the next series of experiments, apoptotic signal transduction events were examined by analyzing caspase-3 activation (Figure 6). Western blot analyses showed that expression of pro-caspase-3 did not change significantly in the treatment groups when compared with 37°C non-treatment control. However, cleaved caspase-3 was slightly increased in the cells treated with 1 hour increased temperature alone. Cleaved caspase-3 was markedly increased in the cells treated with sodium azide/2-DG at both temperatures (37°C and 41°C). Western blot analyses also showed the increase of cleaved product of poly (ADP-ribose) polymerase-1 (PARP-1), a substrate of caspase-3, following these treatments. The increased temperature further elevated sodium azide/2-DG-induced cleavage of PARP-1. Together, these results suggest that caspase signaling pathway was activated by sodium azide/2-DG and increased temperature intensified these signaling events.

**Figure 7:**
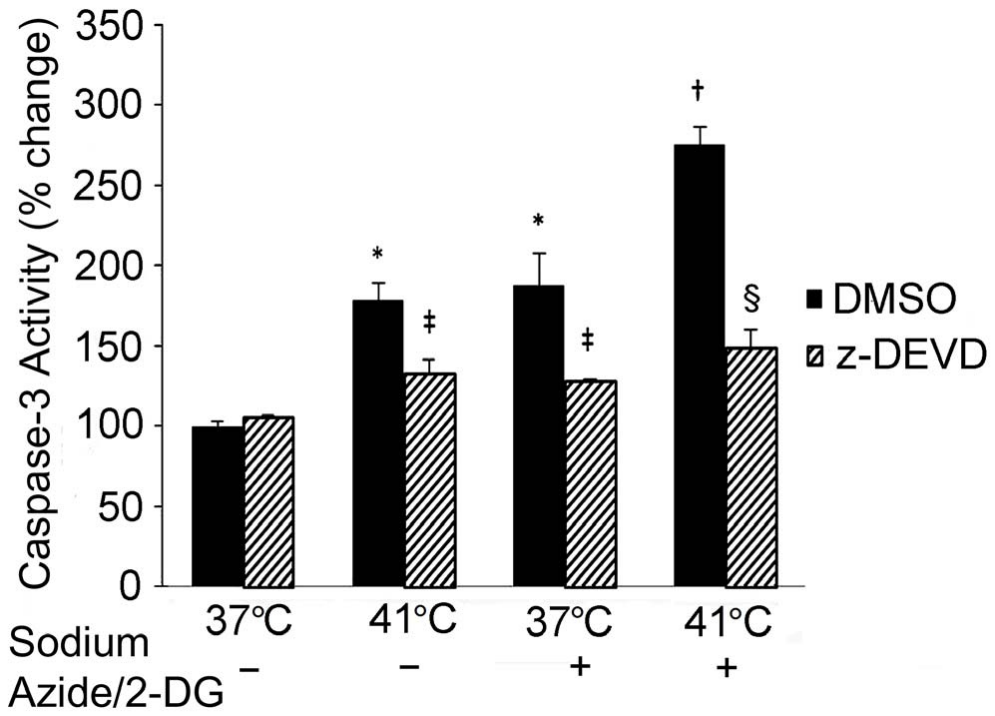
Caspase-3 activity was increased following the treatments. Sodium azide/2-DG, increased temperature or combination of sodium azide/2-DG plus increased temperature resulted in increased caspase-3 activity. Cells were pre-incubated with DMSO or caspase-3 inhibitor (z-DEVD-FMK) for 2 hours, and then cells were subjected to the indicated treatments for 1 hour. * denotes significant difference from the 37°C DMSO cell (*p*<0.01); † denotes significant difference from the 37°C plus sodium azide/2-DG and 41°C alone cells (*p*<0.05); ‡ (*p*<0.05) and § (*p*<0.01) denote significant difference from the DMSO groups, respectively. n = 3.

The activities of caspase-3 were measured to confirm its activation. As seen in Figure 7, treatment with either temperature increase (41°C) or sodium azide/2-DG alone resulted in elevated caspase-3 activities than that in the 37°C DMSO control (*p*<0.01). The combination treatment further elevated caspase-3 activity compared to either sodium azide/2-DG (*p*<0.05) or temperature increase alone group (*p*<0.05). Treatment with the caspase-3 inhibitor, z-DEVD-FMK, significantly reduced caspase-3 activities in temperature increase alone (*p*<0.05), sodium azide/2-DG alone (*p*<0.05) and combination group (*p*<0.01), respectively.

**Figure 8:**
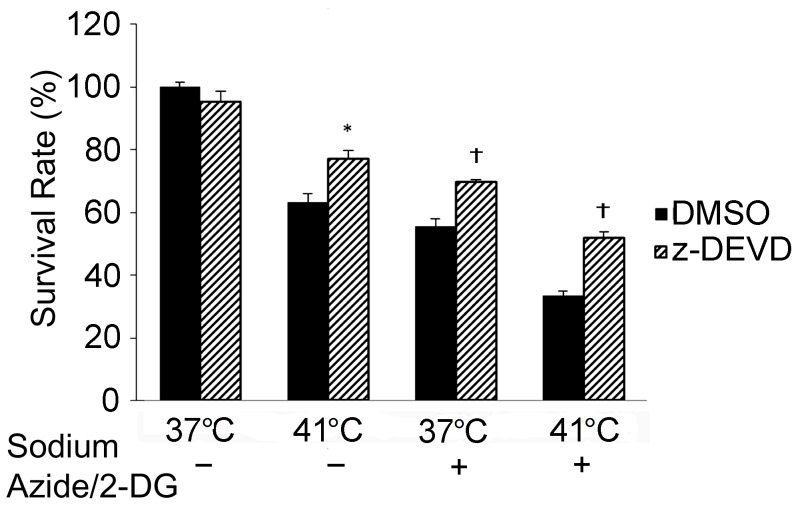
Inhibition of caspase-3 reduced neuronal death following the treatments. Cells were pre-incubated with DMSO or caspase-3 inhibitor (z-DEVD-FMK) for 2 hours, and then cells were subjected to the indicated treatments for 1 hour. Cell death was detected with trypan blue exclusion assay. * (*p*<0.05) and † (*p*<0.01) denote significant difference from the DMSO groups, respectively. n = 4.

Finally, the role of caspase-3 activation in the temperature increase enhanced neuronal death was evaluated. In these experiments, cells were incubated with caspase-3 inhibitor, z-DEVD-FMK, for 2 hours before the treatments. As shown in Figure 8, cells treated with z-DEVD-FMK had significantly higher survival rate than cells treated with DMSO at 41°C without sodium azide/2-DG (*p*<0.05). The inhibitor also significantly increased resistance to sodium azide-induced cell death at 37°C (*p*<0.01) and 41°C (*p*<0.01), whereas z-DEVD-FMK did not significantly affect the viability from DMSO control at 37°C (*p*>0.05).

## Discussion

Although it is clear that temperature increase is deleterious at neuronal levels following ischemic brain injury [3], the mechanisms underlying these deleterious actions are still not clearly defined. To investigate how temperature increase exacerbates ischemic injury processes, in the present study we examined signal transduction pathways using a model of chemical ischemia in neuronal like cells, SH-SY5Y. Similar to a previous report [19], we found that SH-SY5Y cells underwent cell death after chemically induced ischemia in a dose-dependent manner. We also found that a combination treatment of increased temperature and chemically induced ischemia resulted in more cell death than either treatment alone. More importantly, the findings of the present study demonstrated that the combination treatment resulted in more severe intracellular ATP depletion, more intensive ER stress activation and increased apoptotic cell death. These findings reveal new molecular mechanisms underlying temperature increase exacerbated neuronal death during chemical ischemia by regulating signal transduction of ER stress and apoptosis.

In the present study, chemical ischemia was induced by treatment of SH-SY5Y cells with sodium azide and 2-DG. Sodium azide, inhibiting cytochrome c oxidase-respiratory chain complex IV, has been used either alone or in combination with 2-DG, the glycolysis blocker, to induce chemical ischemia *in vitro* and *in vivo* [19], [26]. The mechanisms of this chemical ischemia for the cell death induction include glutamate overflow, calcium overload, free radical production, activation of mitogen-activated protein kinase cascades and apoptotic signal activation, which also play a significant role in injury processes during ischemic injury [20], [27], [28]. Brain function and viability require utilization of ATP derived from the metabolism of glucose. Glucose is oxidized to pyruvate in cytoplasm by enzymes of glycolysis. In the presence of adequate oxygen, pyruvate is further oxidized to CO_2_ and H_2_O in the mitochondria. ATP is produced during this aerobic glucose metabolism, which is the major source of ATP production in the brain. ATP produced by glycolysis may transiently increase to meet sudden demands for metabolic energy in aerobic conditions including stroke. However, ATP production through glycolysis will ultimately fail if the blood flow is not reestablished [29], [30]. Therefore, the model employed here simulated the energy failure due to the interruption of both oxidative phosphorylation and glycolysis. Although in vitro models allow us to study the mechanisms of neuronal death, such studies differ from the animal model of ischemic brain injury by occlusion of one or several major arteries. Furthermore, this *in vitro* model is also different from the physio-pathological conditions in stroke patients. Thus, the findings from the present study may provide important information about the mechanism of neuronal death in response to ischemic injury but more research should be carried out using *in vivo* models.

The injured cells here showed decreased cell volume, which is considered a fundamental and universal characteristic of apoptosis [31]. Concomitantly, the combination treatment worsened the cell volume decrease. These results suggest that the cell death induced was apoptotic. Annexin V staining, which has been widely used in apoptotic cell detection [32], was used to further confirm the type of cell death. Annexin V-FITC binds phosphatidylserine which is extracellularly exposed on the cell surface during apoptosis and accessible due to membrane damage in necrotic cells. Propidium iodide (PI) is excluded by the intact membrane of apoptotic cells, while it is incorporated into the DNA of necrotic cells [33]. Thus, the apoptotic cell death induced by the treatments was further supported by the findings of Annexin V-FITC positive but PI negative cell staining following the treatments.

As the primary energy source in the cell, the ATP supply is of vital importance. Most ATP required by eukaryotic cells is generated by mitochondrial respiration. Sodium azide inhibits the electron transfer between mitochondrial respiratory chain [20] and thus inhibits oxidative ATP production. 2-DG impedes ATP production by glycolysis [34]. In the present study, sodium azide/2-DG as well as temperature increase caused reduced intracellular ATP levels. The combination treatment resulted in more severe ATP depletion than either sodium azide/2-DG or temperature increase alone. Previous studies have revealed that ATP depletion can induce cell death [35], [36]. Together, these findings suggest the more severe ATP depletion could be partly responsible for the increased cell death detected in the combination treated cells.

In rat brain ischemia induced by middle cerebral artery occlusion, ATP release in the stratum is almost double the amount of that in non-ischemic control rats [37]. In the present study, ATP levels in the culture medium at 37°C were decreased while they did not change significantly at 41°C following the chemically induced ischemia. The reasons for these differences are not clear, but it could be due to the different time points chosen for the measurement. In the rat study, the ATP was measured at 220 minutes after ischemia [38], whereas in the present study, ATP was measured at 10 and 20 minutes following ischemia induction. A second possibility is that in the rat model the ATP increase might be due to release from astrocytes [38]. A third possibility is that the severity of neuronal damage in the rat model may be different from that in the model employed here.

During ischemia, ATP depletion impedes the protein folding in the ER and causes unfolded protein accumulation. Furthermore, ATP depletion causes Ca^2+^ release from ER lumen and results in increased Ca^2+^ concentration in the cytosol [39]. Both unfolded protein accumulation and Ca^2+^ disturbance can induce unfolded protein response in the ER [40]. Numerous studies have demonstrated that extensive ER stress causes apoptosis, but the signaling pathways are unclear. There are three main ER stress signal transduction pathways mediated by PERK, IRE1 and ATF6 respectively. CHOP operates as a downstream component of these ER stress pathways [12]. CHOP deficient fibroblasts are resistant to ER stress-induced apoptosis [12] and genetic deletion of PERK results in failure for inducing CHOP [41], [42]. Under normal conditions, PERK is held in an inactive, monomeric state by binding to Bip in ER lumen. ER stress leads to the accumulation of unfolded proteins in the ER lumen, and Bip dissociates from PERK. This dissociation results in PERK auto-activation, which in turn phosphorylates eIF2α. The phospho-eIF2α attenuates general protein translation but enables translation of ATF4. ATF4 promotes cell survival by inducing transcription of anti-apoptotic genes, but it also induces transcription of pro-apoptotic genes, including CHOP gene [14], [43]. A previous study has shown phosphorylation of eIF2α by PERK and increased CHOP in hypoxia-induced neuronal apoptosis [44]. The present study showed that ATP depletion was more severe in 41°C than in 37°C following the treatment with sodium azide/2-DG. Concomitantly, the combination treatment with sodium azide/2-DG and temperature increase elevated eIF2α phosphorylation and CHOP levels more than either treatment alone. Inhibition of CHOP expression by transfection of the cells with shRNA reduced sodium azide/2-DG induced cell death at both 37°C and 41°C conditions. Collectively, these results suggest that activation of the PERK-eIF2α-CHOP pathway was involved in the exacerbation of neuronal cell death by the temperature increase, and that ATP depletion was responsible for its activation. PERK-eIF2α is the first identified molecular mechanism of UPR, and this pathway is activated during ischemic brain injury [15]. Further studies are needed to explore if the other two pathways are also involved in the hyperthermia exacerbated cell death during ischemia.

Studies have found that caspase-3 is activated in cortical neuronal culture following ischemic induction [19] and hypoxic treatment [44]. Activation of caspase family members, including caspase-3, has been observed in ER stress induced apoptosis [44]. The present findings showed that cleaved caspase-3 increased following the treatment with sodium azide/2-DG or increased temperature, suggesting the activation of caspase-3. The caspase-3 activation was also supported by the cleavage of PARP-1, a substrate of caspase-3. Additionally, caspase-3 inhibitor ameliorated the temperature increase and sodium azide/2-DG induced cell death.

Collectively, the findings from the present study suggest that temperature increase exacerbated the neuronal death through depleting ATP, promoting ER stress and activating apoptotic signal transduction in a model of chemically induced ischemia. Further investigation of ischemic neuronal death exacerbated by the temperature increase will shed more light on the molecular mechanisms that regulate neuronal death. Revealing these mechanisms is essential for the development of novel strategies for the treatment of ischemic stroke patients with hyperthermia.
